# Distinct hindlimb motor cortex subregions encode forward and backward hindlimb movements in rats

**DOI:** 10.3389/fnsys.2026.1912148

**Published:** 2026-09-23

**Authors:** Joey St-Arnault, Andrew R. Brown, Marco Bonizzato, Marina Martinez

**Affiliations:** 1 Department of Neurosciences, Centre Interdisciplinaire de Recherche sur le Cerveau et l’Apprentissage (CIRCA), Groupe de Recherche sur la Signalisation Neurale et la Circuiterie, Centre d’Innovation Biomédicale, Université de Montréal, Montréal, QC, Canada; 2 Department of Electrical Engineering and Institute of Biomedical Engineering, Polytechnique Montréal, Montréal, QC, Canada; 3 Mila - Québec AI Institute, Montréal, QC, Canada; 4 CIUSSS du Nord-de-l’île-de-Montréal, Montréal, QC, Canada

**Keywords:** hindlimb, long-duration stimulation, motor control, motor cortex, motor maps, rats

## Abstract

**Introduction:**

The motor cortex is organized to coordinate voluntary movements through distinct subregions that control different parts of the body. Intracortical microstimulation (ICMS) with long-duration trains (LD-ICMS) can uncover complex, behaviorally relevant movement patterns that differ from the simple, joint-specific responses evoked by conventional short-duration trains (SD-ICMS). While previous studies have primarily examined forelimb representations, the organization of hindlimb movements remains less understood.

**Methods:**

Here, we characterized hindlimb movements evoked by LD-ICMS, analyzing their kinematic features, cortical location and functional relevance in rats.

**Results:**

Two distinct complex movements, Advance and Elevation, were identified, each with unique kinematic signatures. Principal component analysis revealed that overall movement amplitude and joint flexion patterns underlying movement trajectory systematically distinguished the two movement types. Advance and Elevation movements were spatially segregated within anteromedial and posterolateral cortical subregions, respectively. Phase-coherent stimulation of these subregions during locomotion selectively biased hindlimb trajectories forward or backward in an amplitude-dependent manner.

**Discussion:**

These results demonstrate that the rat hindlimb motor cortex contains distinct subregions specialized for directionally specific and functionally relevant movement control.

## Introduction

The motor cortex plays a central role in generating voluntary movements. Early studies used electrical stimulation to first examine its intrinsic role (Ferrier and Burdon-Sanderson, 1875; Fritsch and Hitzig, 1870). Intracortical microstimulation (ICMS) has since become a standard tool to map motor representations. Short-duration ICMS trains (<50 ms; SD-ICMS) elicit brief muscle twitches across species, revealing a somatotopic mapping of body musculature in primates (Andersen et al., 1975; Asanuma and Rosén, 1972; Donoghue et al., 1992; Penfield and Boldrey, 1937; Woolsey et al., 1952; Yao et al., 2013) and rodents (Brown and Martinez, 2018; Donoghue and Wise, 1982; Martinez et al., 2009, 2010; Neafsey et al., 1986; Tennant et al., 2012; Young et al., 2011). However, fine-grained somatotopic organization within a single body region is less apparent: while forelimb and hindlimb regions can be distinguished, muscle representations within a single body part are intermixed, and the same body part may be represented at multiple overlapping sites (Luppino et al., 1991; Schieber, 2001). SD-ICMS typically recruits simple neuromuscular units, failing to capture the complexity of coordinated, multi-joint movements involved in natural behavioral movement.

In contrast, long-duration pulse trains (100–500 ms; LD-ICMS) delivered on behaviorally relevant timescales evoke complex, coordinated movements of one or more body parts toward specific postures in primates (Gharbawie et al., 2011; Graziano et al., 2002, 2005; Haiss and Schwarz, 2005; Overduin et al., 2012) and rodents (Bonazzi et al., 2013; Brown et al., 2023; Brown and Teskey, 2014; Halley et al., 2020; Harrison et al., 2012). In primates, LD-ICMS produces ethologically relevant actions such as bringing the hand to the mouth with coordinated jaw opening, reaching, grasping, defensive postures, or climbing movements (Graziano et al., 2002). These actions converge toward stereotyped end-positions, regardless of initial limb configuration, reflecting coordinated activation of multiple muscles and joints. Similar results have been reported in rodents, where LD-ICMS evokes complex forelimb movements such as grasping, reaching, and retraction (Brown and Teskey, 2014; Ramanathan et al., 2006). Like the simple responses to SD-ICMS, these complex movements remain topographically organized across motor cortex.

Despite this progress, most LD-ICMS studies have focused on the forelimb motor cortex, whereas the organization of hindlimb representations remains poorly understood. A recent study showed that LD-ICMS can also elicit complex hindlimb movements in rats, including hip extension driving backward movement (Halley et al., 2020), suggesting that hindlimb motor areas may also be organized to generate complex, ethologically relevant behaviors. However, the internal organization of hindlimb motor representations, and their functional relevance, remains unexplored. By applying LD-ICMS systematically across the hindlimb cortex combined with quantitative kinematic analysis and PCA, we can objectively classify evoked movements, map their cortical distribution, and test their functional relevance during active locomotion, approaches that have not previously been applied to the rat hindlimb motor cortex.

Recent recordings in non-human primates showed that the leg area of the primary motor cortex uses distinct low-dimensional subspaces to simultaneously maintain ongoing locomotor-related dynamics and encode volitional gait adjustments during obstacle avoidance (Xing et al., 2022), suggesting that the cortex implements high-level directional commands without disrupting underlying spinal locomotor rhythms. Whether analogous subregional specialization exists in the rodent hindlimb cortex, and whether such cortical commands can selectively bias ongoing locomotion, remains unknown.

The present study addresses these gaps by characterizing complex hindlimb movements evoked by LD-ICMS in rats and examining their cortical organization. Using kinematic analyses and principal component analysis (PCA), we identified two distinct classes of hindlimb movements, Advance and Elevation, each with unique kinematic signatures and segregated cortical subregions. Phase-coherent stimulation during treadmill locomotion reproduced these movements, selectively biasing hindlimb trajectories forward or backward in an amplitude-dependent manner. Together, these findings show that the rat motor cortex contains discrete hindlimb clusters that encode directionally specific control of locomotion, identifying them as promising targets for neuroprosthetic interventions.

## Materials and methods

### Animals and experimental approach

All procedures followed the guidelines of the Canadian Council on Animal Care and were approved by the Comité de déontologie de l’expérimentation sur les animaux (CDEA; animal ethics committee, protocol #15-125) at Université de Montréal. Thirteen adult female Long-Evans rats weighing 250–330 g (Charles River Laboratories, line 006) were used. Eight rats underwent terminal ICMS experiments to map hindlimb motor representations in one hemisphere. An additional five rats were used to assess the effects of ICMS during treadmill locomotion at 23 cm/s. Rats were housed with ad libitum access to food and water throughout the study.

### Electrophysiological mapping procedures

LD-ICMS was performed following established protocols (Bonazzi et al., 2013; Brown and Teskey, 2014). Rats were anesthetized with ketamine hydrochloride (80 mg/kg, i.p.) and xylazine (5 mg/kg, i.p.) and secured in a stereotaxic frame. Supplemental alternating injections of either ketamine (25 mg/kg) or a mixture of ketamine (17 mg/kg) and xylazine (2 mg/kg) were administered as required to maintain anesthesia, monitored by vibrissae whisking, breathing rate, and foot/tail reflexes in response to gentle pinching. An 8 × 5 mm craniotomy was performed over the right sensorimotor cortex, approximately extending from 3 mm anterior to 5 mm posterior to bregma and from midline to 5 mm lateral. A small puncture was made in the cisterna magna with an 18-gauge needle to reduce cortical edema. Dura was removed, and body-temperature mineral oil was applied to cover the cranial window. A 32× image of the exposed brain was captured with a digital camera (ac-A1300-200 μm, Basler) coupled to a Stemi 2000-C stereomicroscope (Carl Zeiss) and displayed on a computer. A 500 μm grid aligned to bregma was overlaid using Canvas imaging software (v11, ACD Systems). Penetrations were performed at grid intersections, with an interpenetration distance of 354 μm. When an intersection coincided with a blood vessel, the penetration was displaced to the nearest accessible site.

Glass-coated platinum/iridium microelectrodes (0.5 ± 0.1 MΩ, 1,000 Hz, 10 nA; FHC) were used, and impedance was monitored continuously, discarding electrodes below 0.3 MΩ. Electrodes were guided into the neocortex to a depth of 1,550 μm via a microdrive (David Kopf Instruments model 2650), corresponding to layer V pyramidal neurons (Brown and Martinez, 2018, 2021; Teskey et al., 2002). Electrical stimulation was delivered with an isolated pulse stimulator (Model 2100, A-M Systems) through biphasic cathodal-lead pulses (200 μs duration, 333 Hz frequency) for 500 ms at 0.5 Hz. Current intensity was set at 150 μA based on pilot data establishing this value as the optimal intensity. Ground lead was placed on the exposed neck musculature.

The rat trunk was positioned on a polystyrene block with a posterior cutout to allow hindlimb movement and the abdomen supported by a heating pad. Kinematic markers were placed on the iliac crest, greater trochanter, patella, medial malleolus, metatarsals, and toe phalanges. Evoked movements were recorded using a high-speed camera (119 Hz, ac-A1300-200 μm, Basler), synchronized to the stimulation via a visible LED. Any penetration site that failed to elicit a movement at this maximum intensity was considered nonresponsive. Each site was stimulated up to five times, and nonresponsive sites were recorded accordingly. Hindlimb map borders were defined using non-hindlimb movements (neck, jaw, vibrissae, trunk, tail, forelimb) or non-responsive sites, after which the central hindlimb map was filled.

Evoked movements were classified as complex (multi-joint flexion/extension) or simple (single-joint) movements. Anesthesia was monitored every 15 min by verifying thresholds at previously defined positive-response sites. A light-emitting diode (LED) synchronized with the stimulator output was placed within the camera field-of-view to mark stimulation timing. Each responsive site was considered to represent 0.125 mm^2^ of cortical surface (354 × 354 μm). Map organization was assessed by calculating the total cortical area representing hindlimb movements, the proportion of complex versus simple movements, and the representation of individual hindlimb joints.

Complex movements were initially classified as Advance or Elevation by visual inspection of video recordings by two independent observers, based on the direction of limb displacement and joint recruitment sequence. To validate these classifications objectively, PCA was subsequently performed blind to the initial labels. The PCA independently recovered the same two-category structure (Figure 6A), confirming the validity of the initial classification. We also acknowledge that some multi-joint responses may partly reflect the mechanical consequences of activating biarticular muscles rather than the recruitment of independent coordinated programs. However, the convergence of evoked movements to stereotyped end-postures regardless of initial limb configuration, and the objective confirmation by PCA of two kinematically distinct categories (Figure 6), support the interpretation that these represent coordinated motor programs rather than simple mechanical responses to single-muscle activation.

#### Kinematics analysis of complex movements

For each stimulation site, videos included multiple trials. Clips were extracted including 10 frames before the LED onset (baseline) until movement cessation. Videos were analyzed using DeepLabCut (Mathis et al., 2018), a markerless pose estimation deep learning algorithm capable of tracking different body parts using deep neuronal networks. For network training, three videos per rat were used, and 20 frames per video were extracted using a k-means algorithm to cluster frames by visual appearance. Frames were marked for the following joints: iliac crest, hip, knee, ankle, metatarsophalangeal joints (MTP), and toe. The network was trained to estimate the X and Y positions of these joints in each frame, using a total of 480 manually labeled frames from 24 videos. After 300,000 iterations, the network achieved a mean error of 1.72 pixels between predicted and manually labeled points, demonstrating accurate tracking. All videos were then analyzed to extract coordinates for all six points.

Kinematic measures were calculated independently for each point in the X and Y axes (Bonazzi et al., 2013). Multiple trials were recorded per stimulation site; the first trial was used for analysis, with others as backups. Instantaneous speed (mm/s) was calculated as the tangential derivative of position over time, and maximal instantaneous speed (mm/s) was determined. Movement onset was defined as the frame when instantaneous speed exceeded 5% of the maximal observed speed. Latency was defined as the interval from stimulation onset to movement onset. Movement termination was defined as the position reached at the end of the 500 ms stimulation. For each joint, multiple variables were calculated. Mean speed was calculated by calculating overall distance travel from initial position to maximal position using Euclidean distance from each coordinate divided by the time to reach the maximal position. Maximal speed was calculated by calculating Euclidean distance between two consecutive points and divide this value by time which correspond to the frame rate of the camera. The maximal value corresponded to maximal instant speed. Total trajectory was calculated by calculating the summation of all Euclidean distance from 0 to 500 ms. The vector length was calculated by calculating the Euclidean distance from initial to final position reached at 500 ms. The angle variation of each joint was calculated by calculating the tangent report of each joint, then by adding or subtracting adjacent joints to obtain joints angle variation. The angle variation was the difference between the angle at the end of the movement at 500 ms and the angle at initial position.

#### Typological and topographic analysis

Typological analysis of complex movements was performed using principal component analysis (PCA) after standardizing the following kinematic features: maximal X and Y displacement, time to reach maximal position, mean speed, maximal instantaneous speed, latency, movement duration, vector magnitude and angle from initial to final position, trajectory distance, and hip, knee, ankle, and MTP angle variation.

For topographic analysis of ICMS maps, the centroid of each complex movement was calculated. A line was drawn connecting the centroids, with its center defined as zero. Each stimulation site coordinate was projected onto this line, with antero-medial projections assigned negative values and posterior-lateral projections positive values. Student’s *t*-tests were used to compare projection scores between complex movements. Projection scores were plotted against PCA scores to visualize the relationship between movement kinematics and cortical location.

### Awake experiments

Intracortical microstimulation experiments in awake, behaving rats were conducted following procedures described in our previous studies (Bonizzato and Martinez, 2021; Drainville et al., 2025; Massai et al., 2023).

#### Electrode implantation

All surgical procedures were conducted under general anesthesia with isoflurane. To enable real-time detection of the gait cycle and phase-specific stimulation, bipolar intramuscular electrodes were implanted in the left and right tibialis anterior (ankle flexor) and medial gastrocnemius (ankle extensor) muscles. The exposed wire tips created an interface for EMG recording. A craniectomy was performed over the right hindlimb motor cortex, and a 3 × 2 mm section of dura was removed to allow insertion of a 32-channel intracortical microelectrode array (Tucker-Davis Technologies). The array consisted of four columns spaced 0.375 mm apart and eight rows spaced 0.250 mm apart and was stereotaxically lowered to target layer V of the cortex (depth: 1.5 mm). The anteromedial corner of the array was aligned to coordinates (posterior: 1 mm, lateral: 1.3 mm) relative to bregma. The array and EMG connector were secured using dental acrylic and five micro-screws. Postoperative care included antibiotics and analgesics for 3–4 days.

#### Behavioral procedures

Rats were trained with positive reinforcement to walk on a motorized treadmill at a constant speed of 23 cm/s. During recording sessions, six reflective markers were placed on anatomical landmarks (iliac crest, hip, knee, ankle, foot, and toe tip), and kinematics were captured at 119.2 Hz. Each trial consisted of 10 consecutive steps under stable locomotor conditions. Marker trajectories were tracked using DeepLabCut (Mathis et al., 2018) and manually curated to correct misidentifications. Automated gait analysis was then used to extract kinematic parameters to assess locomotor performance. Step height was calculated as the difference between the average vertical position of the foot during stance and its maximum vertical displacement during swing.

#### Phase-coherent ICMS during locomotion

Electromyographic (EMG) signals were acquired at 6 kHz and preprocessed online using a BioAmp system (Tucker-Davis Technologies). Signals were bandpass filtered (70–700 Hz), rectified, and monitored in real time. When the rectified activity of the selected ankle flexor crossed a predefined threshold, a gait event was detected, marking the onset of muscle activation. A 350-ms refractory window followed each detection to avoid multiple triggers within a single step. Detected events triggered ICMS with a 40-ms delay. Stimulation consisted of a 40-ms train of biphasic pulses (330 Hz, cathodic first, 200 μs per phase, 50-μs interphase interval). One stimulating channel was selected within each functionally identified cortical region, based on the strongest response observed during passive testing. Stimulation amplitude was adjusted within a functional range: the lower bound corresponded to the minimal visible movement change (40–100 μA), while the upper bound reached approximately 90% of the maximum intensity tolerated by the rat during treadmill walking (80–300 μA). This upper limit was determined behaviorally, based on the animal’s continued engagement with the task while receiving stimulation and motivated by food rewards.

### Statistical analysis

For each statistical test, data distribution was assessed for normality using the one-sample Shapiro-Wilk test. For kinematic analyses under anesthesia (Figure 3), Mann-Whitney U tests were applied to each marked joint to compare between complex movement types (Advance and Elevation). No correction for multiple comparisons was applied to the kinematic analyses (Figure 3), as each joint comparison was pre-specified based on anatomical identity rather than selected *post hoc*. This approach is consistent with treating each of the six joints as an independent, anatomically motivated outcome measure. For cortical mapping data (Figure 4), paired two-tailed Student’s *t*-tests were conducted to assess differences in cortical representation across movement types (simple vs. complex; Advance vs. Elevation). For comparison of projection value onto the line between centroid (Figure 5), Mann-Whitney U test was applied between projection value of Advance and Elevation movement. For comparison of PC2 score (Figure 6), Mann-Whitney U test was applied between PC2 score of Advance and Elevation movement. Effect sizes for unpaired and paired comparisons were calculated using rank-biserial correlation r for Mann-Whitney U tests and Cohen’s dz for paired *t*-tests, and 95% confidence intervals were computed for mean differences. PCA was performed in Matlab (pca function) after data standardization by calculating Z score. For the final kinematic analysis on locomotion (Figure 8), paired Student’s *t*-tests were applied comparing max and no stimulation. Tests were one-sided because stimulation was hypothesized to strictly increase step height. The nonparametric Wilcoxon signed-rank test was used to compare vector angles between Advance and Elevation movements. A one-sided test was used based on the hypothesis that Advance movements would be more forward-positioned than Elevation movements.

Statistical significance was set at α = 0.05. Significance levels are reported as *p < 0.05, **p < 0.01, and ***p < 0.001. Statistical analyses were performed using SPSS Statistics 29 or MATLAB.

## Results

### Hindlimb movements evoked by LD-ICMS

A total of 501 cortical sites were stimulated with LD-ICMS in eight Long-Evans rats, of which 227 sites evoked hindlimb movements. Unlike SD-ICMS, which elicited only simple, single-joint twitches, LD-ICMS produced both simple and complex multi-joint movements. Six simple movements were identified: flexion of the hip, knee, ankle, and toes, as well as extension of the hip and ankle.

In addition, two distinct complex movements were consistently observed. *Advance* movements consisted of simultaneous flexion of the hip and ankle that lifted the hindlimb forward from its baseline semi-flexed posture (Figure 1A). *Elevation* movements began with hip and knee flexion, elevating the hindlimb to a peak position, followed by hip extension and slight backward displacement to a final movement endpoint (Figure 1B).

**FIGURE 1:**
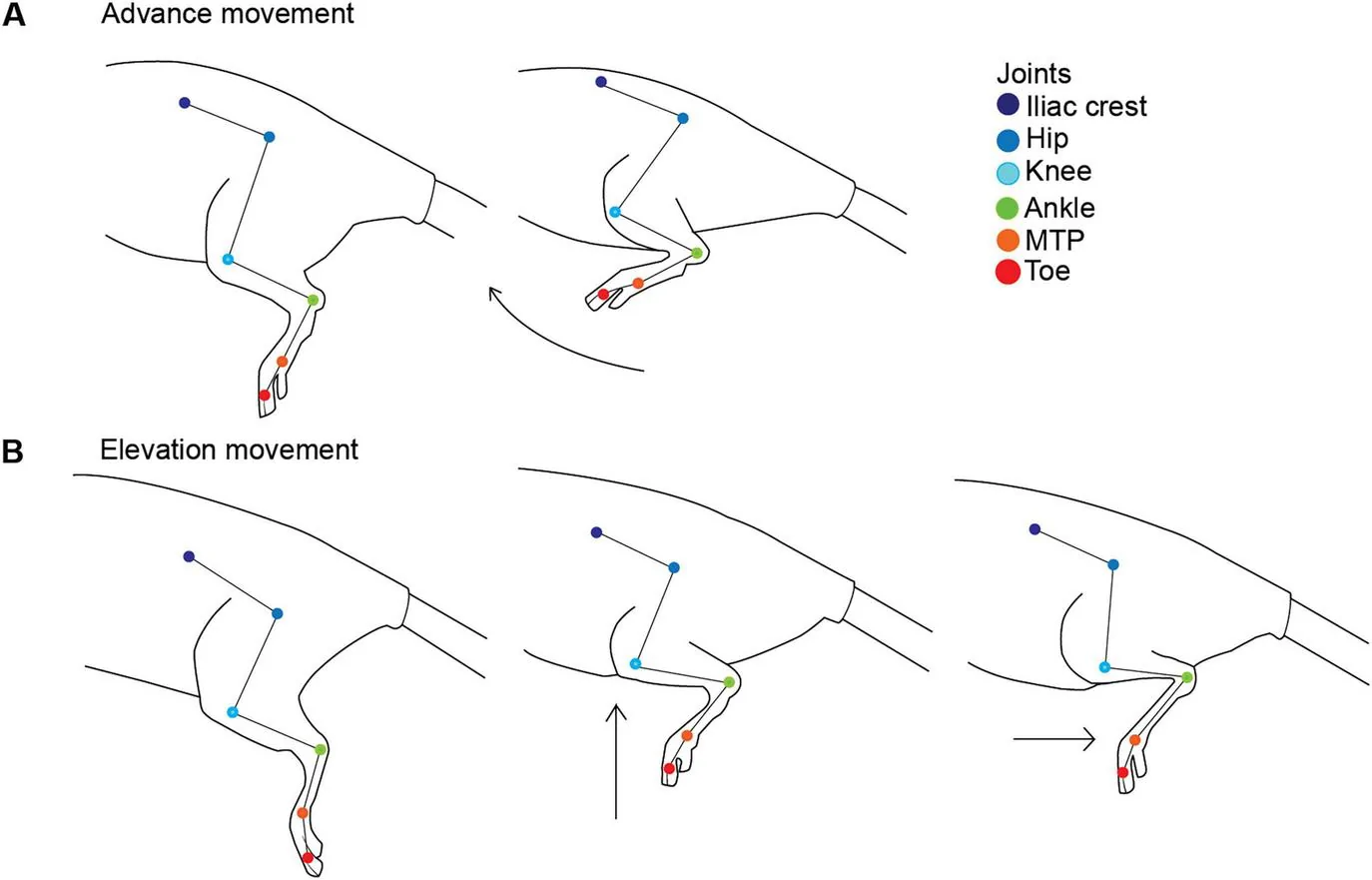
Kinematics of complex movements evoked by LD-ICMS. **(A)** Advance movement. **(B)** Elevation movement. Stick representation representing joint from baseline to final position.

### Effects of stimulation parameters

To assess the role of stimulation parameters, two sites were tested across a range of durations and intensities. Increasing the train duration from 39 ms, the standard for SD-ICMS, up to 1,000 ms (at constant 100 μA intensity) progressively recruited additional joints, transforming simple movements into complex ones. Durations longer than 500 ms did not further alter displacement but maintained the hindlimb at its maximal movement endpoint until the end of the train (Figure 2A).

**FIGURE 2:**
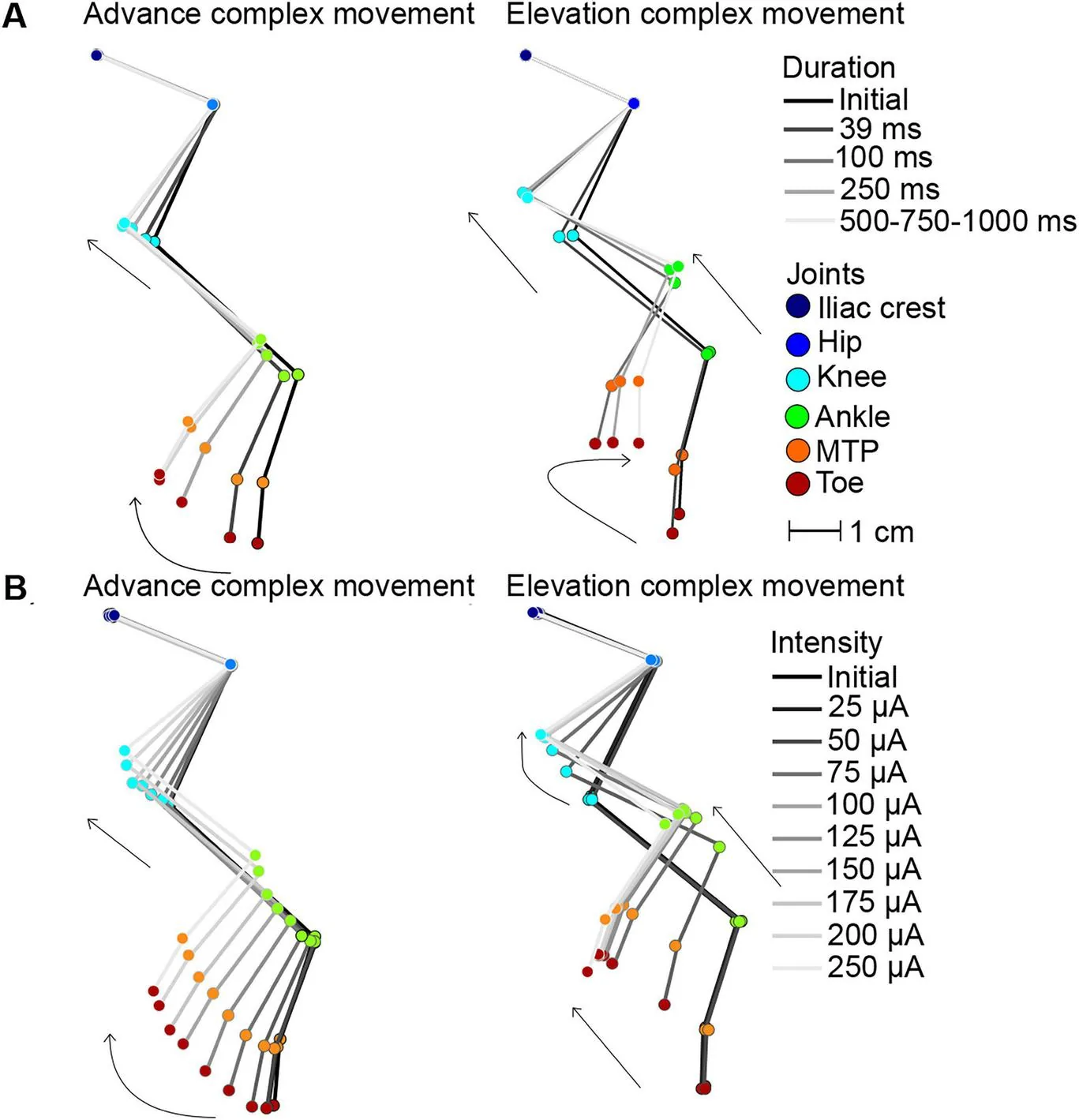
Effects of stimulation duration and intensity on hindlimb kinematics. **(A)** Joint displacement across increasing stimulation duration at constant intensity (100 μA). **(B)** Joint displacement across increasing stimulation intensities at constant duration (500 ms).

Increasing stimulation intensity from 25 to 250 μA at a constant duration of 500 ms increased the amplitude of joint displacements, driving the hindlimb progressively further away from its baseline position (Figure 2B).

Together, these observations from two representative sites suggest that stimulation duration primarily determines the number of joints recruited, driving the transition from simple to complex movements, whereas stimulation intensity regulates the amplitude of the resulting movement. While based on a limited sample, the pattern was consistent across both tested sites.

### Kinematic analysis of complex movements

For each stimulated site at 150 μA for 500 ms, videos were recorded and analyzed to extract six kinematic parameters: latency, movement duration, mean speed, maximal instantaneous speed, vector length, and trajectory length across the six tracked joints. Comparisons between Advance and Elevation movements revealed systematic differences.

Latency was significantly longer at the knee for Advance (0.069 ± 0.030 s) compared to Elevation (0.056 ± 0.030 s; U = 4313.5, *p* = 0.023; Figure 3A), indicating a slightly delayed joint recruitment consistent with a more gradual, propulsive movement.

**FIGURE 3:**
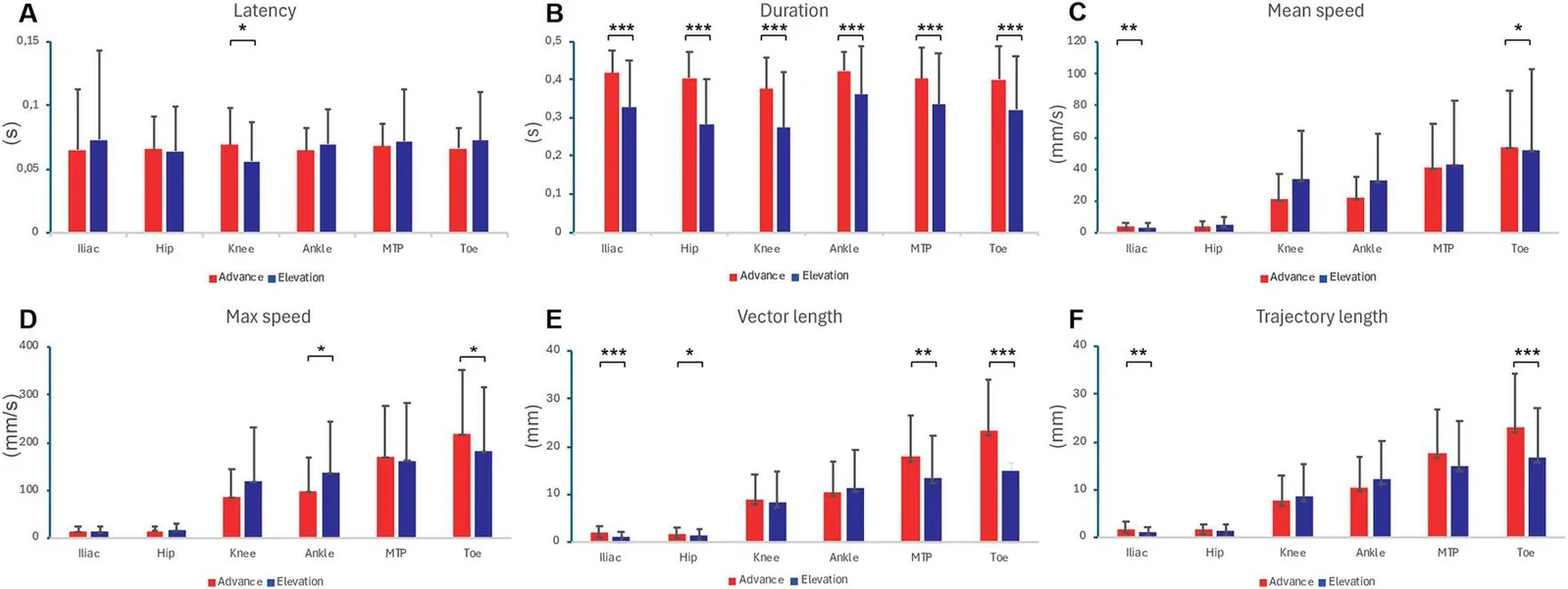
Kinematic parameters of complex hindlimb movements. **(A–F)** Latency **(A)**, duration **(B)**, mean speed **(C)**, maximal speed **(D)**, vector length **(E)**, and trajectory length **(F)** for all joints for Advance and Elevation movements. Data are presented as mean ± SD (Advance: *n* = 101; Elevation: *n* = 71). *P < 0.05, **p < 0.01, ***p < 0.001.

Movement duration was significantly shorter for Elevation compared to Advance movements across all joints (Figure 3B), indicating that Elevation reached its final movement endpoint more rapidly (Iliac: Advance: 0.416 ± 0.060 s; Elevation: 0.326 ± 0.123 s; U = 5359, *p* = 3.43 × 10^–8^, rank-biserial correlation = −0.396; Hip: Advance: 0.403 ± 0.071 s; Elevation 0.282 ± 0.119 s; U = 5713, *p* = 3.63 × 10^–11^, rank-biserial correlation = −0.489; Knee: Advance: 0.376 ± 0.083 s; Elevation: 0.273 ± 0.145 s; U = 4873.5, *p* = 6.19 × 10^–^5, rank-biserial correlation = −0.359; Ankle: Advance: 0.422 ± 0.051 s; Elevation: 0.361 ± 0.125 s; U = 4694.5, *p* = 0.000546, rank-biserial correlation = −0.223; MTP: Advance: 0.402 ± 0.081 s; Elevation: 0.333 ± 0.134 s; U = 4544, *p* = 0.00282, rank-biserial correlation = −0.184; Toe: Advance: 0.400 ± 0.086 s; Elevation: 0.321 ± 0.138 s; U = 4771.5, *p* = 0.000220, rank-biserial correlation = −0.243). Effect sizes were large at proximal joints and moderate at distal joints, with the hip showing the greatest difference (Advance: 0.403 ± 0.071 s; Elevation: 0.282 ± 0.119 s). This rapid endpoint achievement suggests that Elevation is a fast, ballistic action optimized for rapid limb lifting.

Speed analyses further distinguished the two movement types (Figures 3C, D). Advance exhibited significantly higher mean speed at the iliac crest (Advance: 3.9 ± 2.7 mm/s; Elevation: 3.2 ± 3.3 mm/s; U = 4533, *p* = 0.003, rank-biserial correlation = −0.181; Figure 3C). Although mean speed at the toe also differed significantly (U = 4309, *p* = 0.025), the effect was small (rank-biserial correlation = 0.202) and means were largely overlapping (Advance: 54.0 ± 35.9 mm/s; Elevation: 52.3 ± 50.2 mm/s). Regarding maximal speed, Elevation exhibited significantly faster maximal speed at the ankle (Advance: 98.1 ± 71.9 mm/s; Elevation: 135.9 ± 107.2 mm/s; U = 2940, *p* = 0.045, rank-biserial correlation = 0.180; Figure 3D), while Advance exhibited higher maximal speed at the toe (Advance: 218.1 ± 133.2 mm/s; Elevation: 181.6 ± 135.0 mm/s; U = 4232, *p* = 0.045, rank-biserial correlation = −0.180; Figure 3D). These findings suggest that Elevation is characterized by rapid ankle activation consistent with a fast lifting program, whereas Advance generates greater distal velocity consistent with forward propulsion.

In contrast, displacement measures were greater for Advance movements (Figures 3E, F). Vector lengths were significantly larger for the Advance movement at the iliac crest (Advance: 1.9 ± 1.3 mm; Elevation: 1.1 ± 1.0 mm; U = 4997, *p* = 0.0000114, rank-biserial correlation = −0.301; Figure 3E), hip (Advance: 1.8 ± 1.2 mm; Elevation: 1.4 ± 1.2 mm; U = 4469, *p* = 0.006, rank-biserial correlation = −0.164; Figure 3E), MTP (Advance: 17.8 ± 8.9 mm; Elevation: 13.4 ± 8.9 mm; U = 4645, *p* = 0.00099, rank-biserial correlation = −0.210; Figure 3E), and toe (Advance: 23.2 ± 10.9 mm; Elevation: 15.0 ± 10.0 mm; U = 5124, *p* = 0.00000172, rank-biserial correlation = −0.335; Figure 3E). Trajectory lengths were also greater for Advance movements at iliac crest (Advance: 1.8 ± 1.4 mm; Elevation: 1.2 ± 1.1 mm; U = 4631, *p* = 0.00115, rank-biserial correlation = −0.207; Figure 3F), MTP (Advance: 17.7 ± 9.1 mm; Elevation: 14.9 ± 9.6 mm; U = 4285, *p* = 0.0297, rank-biserial correlation = −0.116; Figure 3F) and toe (Advance: 23.1 ± 11.3 mm; Elevation: 16.8 ± 10.4 mm; U = 4776, *p* = 0.000215, rank-biserial correlation = −0.244; Figure 3F). These results indicate that Advance movements involve larger joint excursions across the limb, with moderate-to-large effect sizes at proximal and distal joints.

Together, these results indicate that Elevation movements are faster and reach their movement endpoint more quickly, whereas Advance movements produce larger displacements and longer trajectories. The joint-specific differences, with consistently medium-to-large effect sizes for duration and displacement, suggest a functional specialization, with Elevation movements optimized for rapid limb lifting and Advance movements optimized for rapid forward displacement of the swinging limb during the swing phase of gait.

### Topographic organization of complex hindlimb movements

LD-ICMS evoked contralateral hindlimb movements, with motor maps spanning 0.5 mm anterior to 2.5 mm posterior to bregma and 0.75–3.5 mm lateral to the midline (Figure 4A). Advance movements localized to the anteromedial region, Elevation movements to the posterolateral region, and simple movements were confined to the posterolateral periphery, occupying only a small portion of the map. In contrast, complex movements clustered centrally within the mapped area (Figure 4B).

**FIGURE 4:**
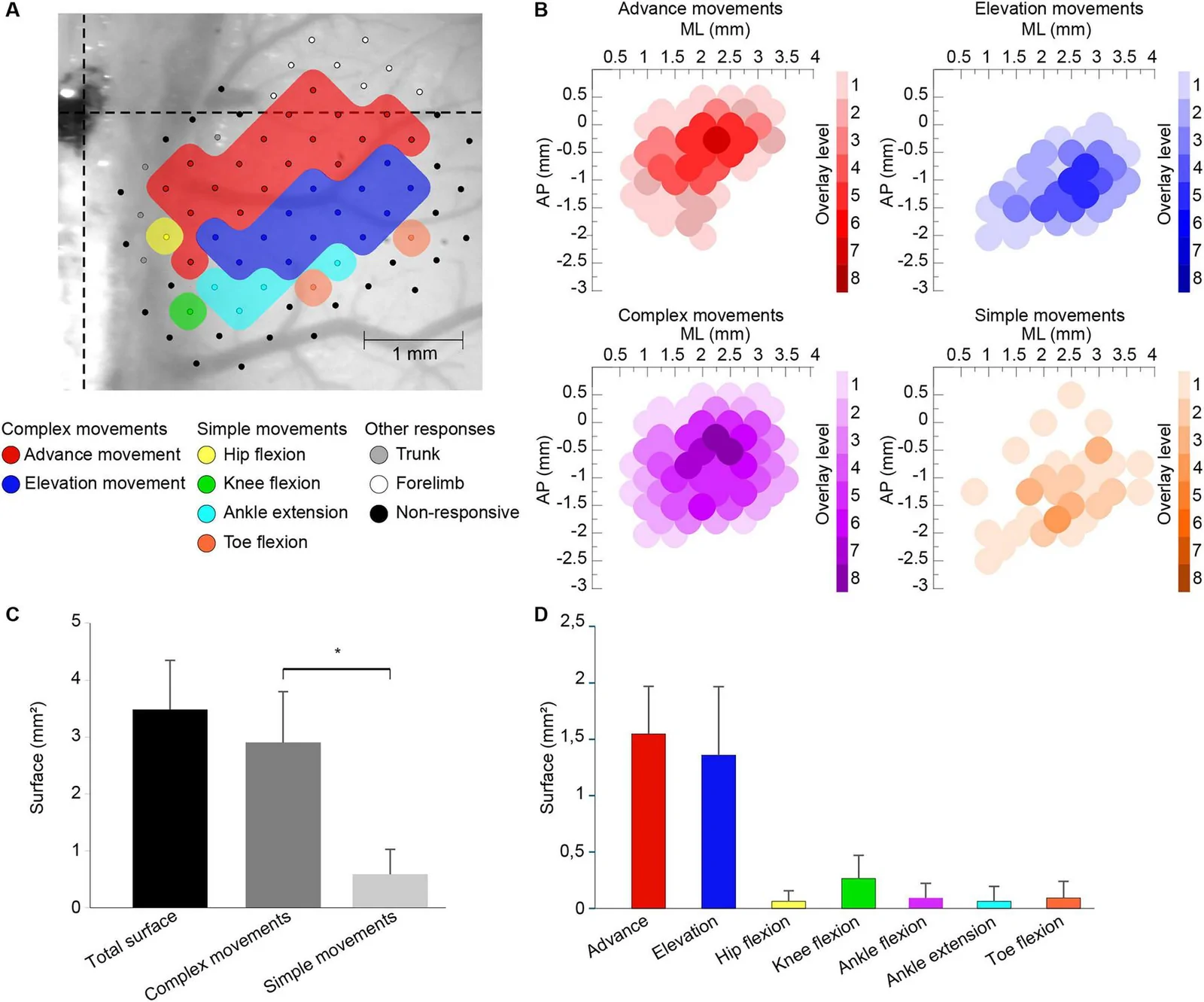
Topographical organization of hindlimb motor maps. **(A)** Representative hindlimb motor map evoked with LD-ICMS (500 ms). **(B)** Surface heatmaps showing the spatial distribution and frequency (overlay level) of evoked movements (*n* = 8). **(C)** Cortical area occupied by complex and simple movements within the hindlimb motor cortex. **(D)** Comparison of cortical area between complex and simple movements. Data are presented as mean ± SD (*n* = 8). *P < 0.05.

The total cortical area that evoked hindlimb movement by LD-ICMS was 3.48 ± 0.87 mm^2^, representing the full spatial extent of evoked responses and comprising both complex and simple movement representations. Complex movements occupied a significantly larger cortical area than simple movements [Complex: 2.91 ± 0.89 mm^2^; Simple: 0.57 ± 0.44 mm^2^; paired *t*-test: *t*(7) = 5.93, *p* = 5.80 × 10^–4^, Cohen’s *d*_*z*_ = 2.10], with a mean difference of 2.33 mm^2^ (95% CI [1.40, 3.26]) (Figure 4C). Advance and Elevation movement areas were comparable (Advance: 1.55 ± 0.42 mm^2^; Elevation: 1.36 ± 0.61 mm^2^; *t*(7) = 0.97, *p* = 0.36) (Figure 4D).

Among simple movements, all five types individually occupied significantly less cortical area than both Advance and Elevation (all paired *t*-tests: *p* ≤ 0.003; Figure 4D). Knee flexion was the most consistently represented simple movement, present in 6 of 8 animals (0.27 ± 0.21 mm^2^; Wilcoxon signed-rank test: W = 21, *p* = 0.016). The remaining simple movement types were sparse and variable across animals: ankle flexion was present in 4/8 rats (0.09 ± 0.13 mm^2^; W = 10, *p* = 0.063), hip flexion in 3/8 (0.06 ± 0.09 mm^2^; W = 6, *p* = 0.125), toe flexion in 3/8 (0.09 ± 0.15 mm^2^; W = 6, *p* = 0.125), and ankle extension in only 2/8 (0.06 ± 0.13 mm^2^; W = 3, *p* = 0.250). These findings indicate that complex movements occupy a dominant proportion of the hindlimb motor cortex, while simple movements are confined to peripheral regions and represent a minor, variable component of the map.

We further analyzed the topography of hindlimb complex movements by calculating the centroids of Advance and Elevation movements (Figure 5A). Each stimulation site was orthogonally projected onto a line connecting the two centroids (Figure 5B), with the line’s midpoint defined as the origin. Projections anteromedial to the origin were assigned negative values, while posterolateral projections were positive (Figure 5C). Projection values differed significantly between Advance and Elevation movements (Advance: −0.377 [−0.718 to −0.097]; Elevation: 0.382 [0.118–0.645]; U = 5466, *p* = 3.34 × 10^–16^, rank-biserial correlation = −0.665), confirming the spatial segregation of these two complex movement types.

**FIGURE 5:**
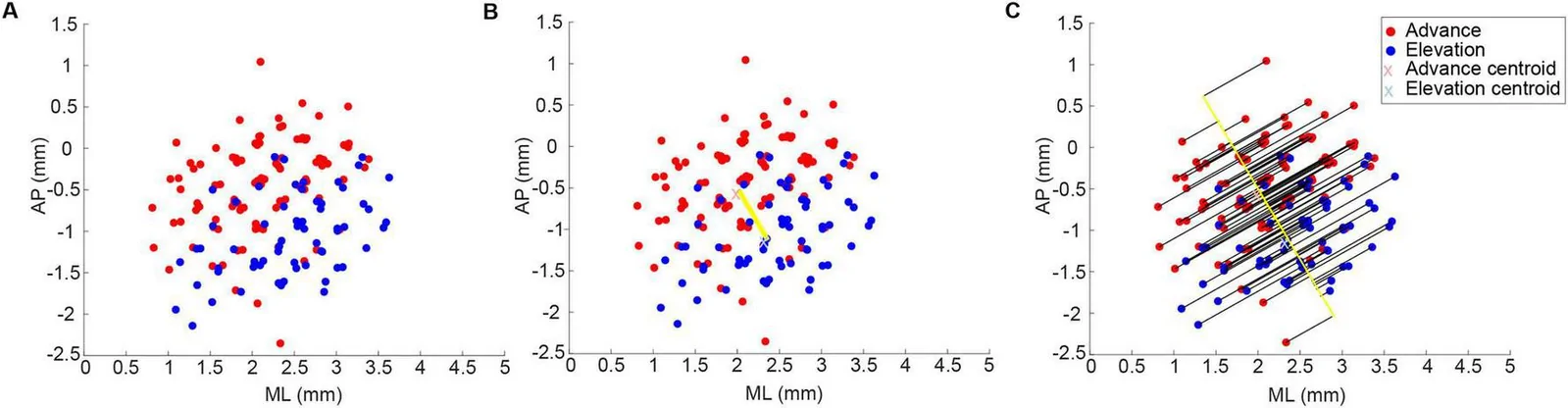
Topographical analysis of complex hindlimb movements. **(A)** Stimulation coordinates for evoked Advance and Elevation complex movements, relative to bregma are shown overlaid. Movements evoked at the same coordinates between animals are shown offset (up to 0.15 mm) for clarity. **(B)** Centroids for Advance and Elevation movements are connected by a yellow line. The midpoint of this line is designated as the origin (0). **(C)** Stimulation coordinates projection onto the centroid line and the distance from midpoint for each projection was calculated. Anteromedial projections have negative values, while posterolateral projections have positive values, providing a quantitative measure of their spatial separation.

### Typological analysis of complex hindlimb movements

We performed a PCA to identify key features distinguishing complex hindlimb movements. Two primary components captured the majority of the data variance. PC1 accounted for 46% of the variance (Figure 6A) and was strongly correlated with overall movement vector amplitude across all joints [r^2^ = 0.96, *F*_(1,160)_ = 3590, *p* < 0.001], reflecting movement size (Figures 6A, B). PC2 explained 17% of the variance and was strongly correlated with relative ankle and knee flexion [r^2^ = 0.69, *F*_(1,160)_ = 356, *p* < 0.001], capturing joint-specific flexion patterns (Figure 6C).

**FIGURE 6:**
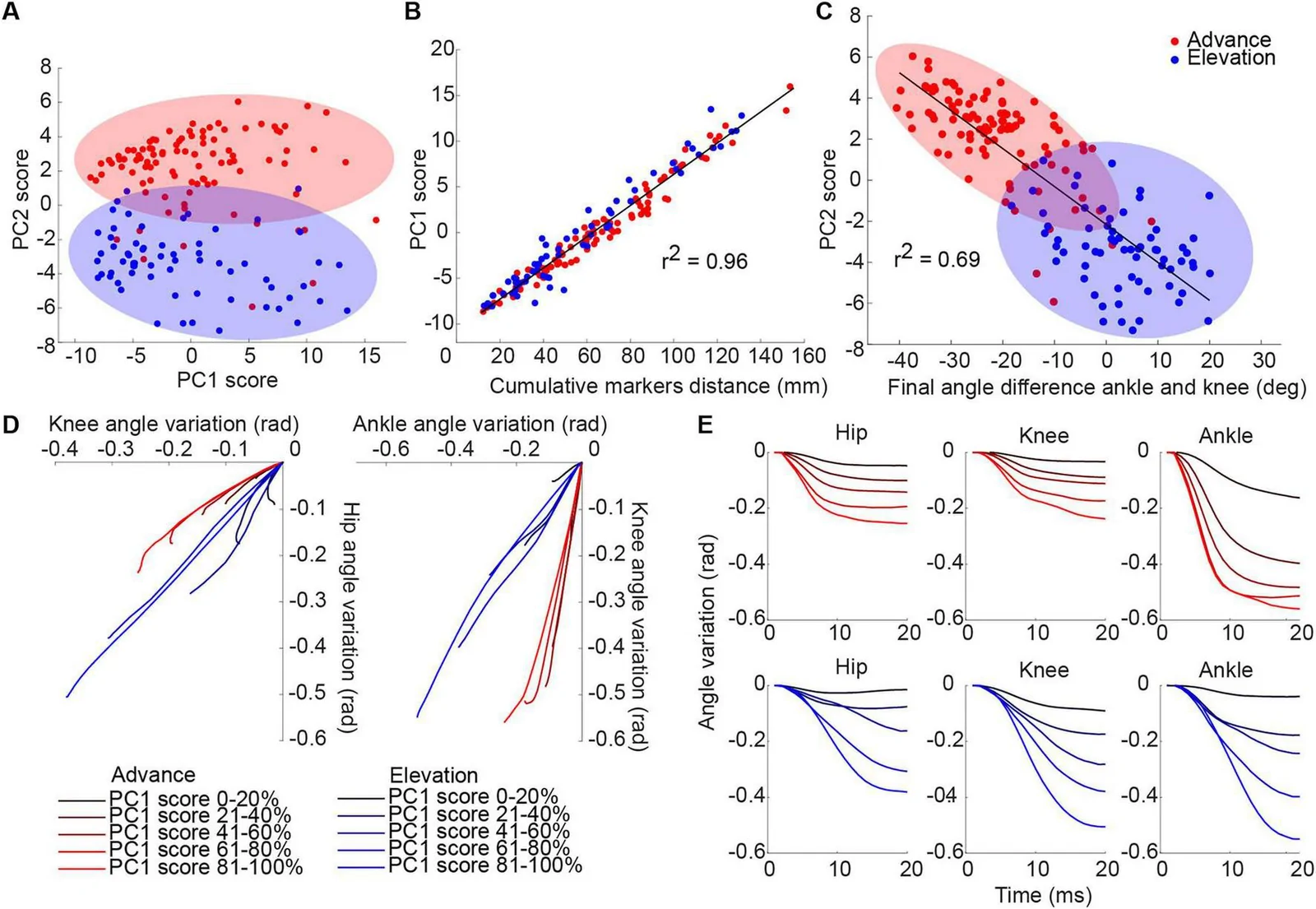
Typological analysis of complex movements. A principal component analysis (PCA) was conducted on kinematic and topographic data with the aim to construct summary indices exploring the data variance between complex movements on these measures. **(A)** Distribution of movements in PC1-PC2 space. **(B)** Correlation between PC1 and movement amplitude. **(C)** Correlation between PC2 and joint angle difference. **(D,E)** Variations in joint angles categorized by specific PC1 score subgroups.

When PC2 scores were plotted against PC1 scores, Advance and Elevation movements formed distinct clusters along the PC2 (Advance: 2.64 [1.45–3.61]; Elevation: −3.38 [−4.54 to −2.34], U = 236, *p* = 2.19 × 10^–22^, rank-biserial correlation = 0.93), indicating that these movement types are primarily distinguished by joint-specific flexion rather than overall amplitude. Further analysis using PC1 scores to classify movements into groups of increasing amplitude revealed systematic differences in joint contributions (Figures 6D, E). Advance movements exhibited greater ankle flexion relative to knee flexion, with faster ankle closure, whereas Elevation movements showed faster and more pronounced knee flexion with reduced ankle contribution. These findings highlight the functional specialization of complex hindlimb movements.

### Relationship between complex movement topography and typology

We next examined how movement typology relates to cortical organization. Overlaid heatmaps across animals revealed that Advance movements clustered in the anteromedial region of the hindlimb motor cortex, while Elevation movements localized to the posterolateral region, with overlap mainly at the map borders (Figure 7A). This spatial segregation indicates that the two types of complex movements are controlled by distinct cortical subregions.

**FIGURE 7:**
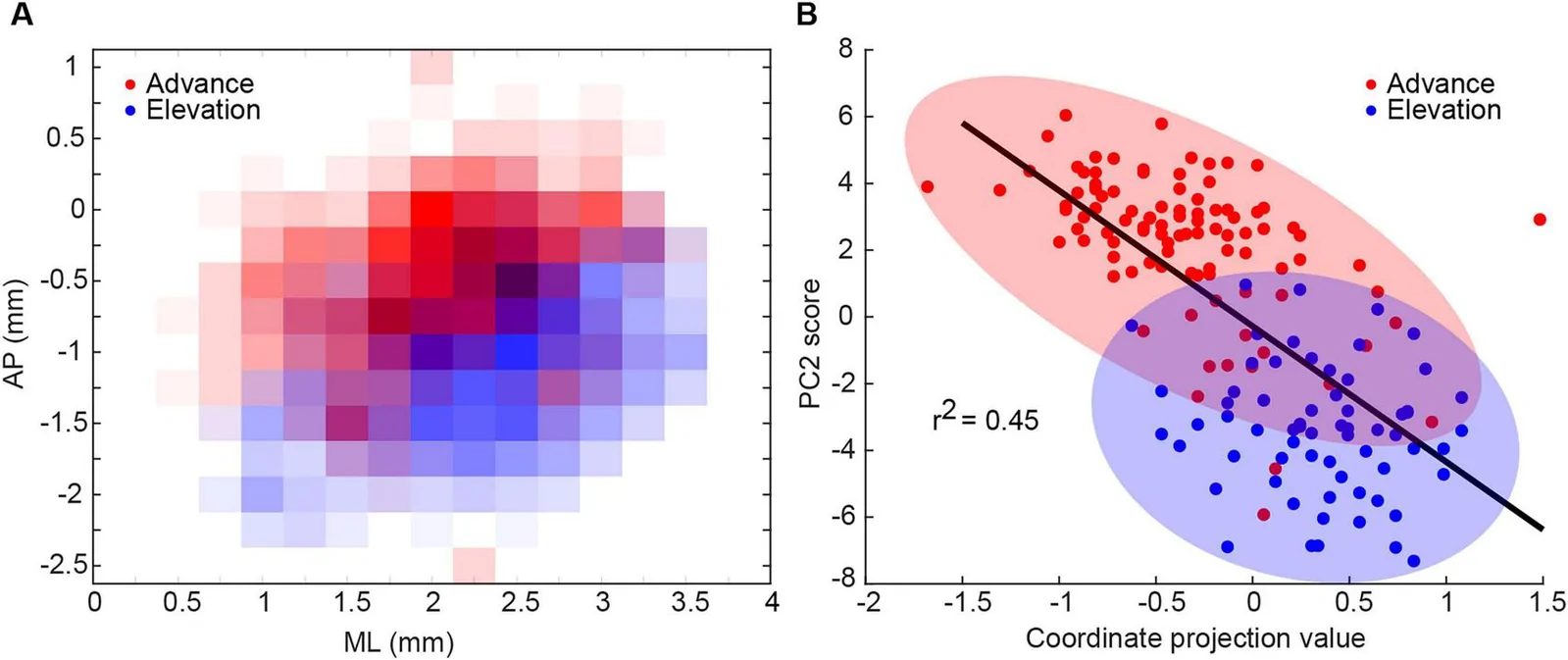
Relationship between complex movements topography and typology. **(A)** Spatial distribution of Advance and Elevation movements (*n* = 8). **(B)** Relationship between projection along centroids axis and PC2 scores.

To quantify this relationship, PC2 scores were plotted against centroid line projections of each stimulation site along the anteromedial-to-posterolateral axis (Figure 7B). Advance and Elevation movements formed distinct spatial clusters, with a significant correlation between PC2 scores and projection values [r^2^ = 0.45, *F*_(1,160)_ = 130, *p* < 0.001]. This analysis demonstrates a strong correspondence between movement typology (PC2, joint flexion patterns) and cortical topography (anteromedial vs. posterolateral localization).

### Stimulation of the two hindlimb cortical subregions distinctly modulates gait kinematics

To test whether the spatially segregated cortical subregions controlling Advance and Elevation movements differentially modulate gait, we applied phase-coherent ICMS during treadmill locomotion in *N* = 5 rats. Stimulation of sites in the forward- (Advance-related) and backward- (Elevation-related) movement subregions produced markedly divergent foot trajectories relative to spontaneous gait. Forward ICMS biased foot motion upward and anteriorly, consistent with the kinematic profile of Advance movements, whereas backward ICMS increased step height and redirected movement posteriorly, reflecting the characteristics of Elevation movements (Figures 8A–C and Supplementary Video 1). These effects scaled with stimulation amplitude {step height increase in max vs. no stimulation +130 ± 29% [*t*(4) = 8.31, *p* = 0.0006] for Elevation and +70 ± 17% [*t*(4) = 5.78, *p* = 0.002] for Advance} and were consistent across animals (Figure 8D). Vector-analysis quantification of peak foot displacement confirmed the directional bias induced by each stimulation site (Figures 8E, F). The vector angle difference, at max amplitude, was 41 ± 10 degrees (W = 15, *p* = 0.03). Together, these results demonstrate that the functional specialization of cortical subregions for different complex hindlimb movements translates directly into selective modulation of gait kinematics in behaving animals, linking cortical topography and movement typology to motor output.

**FIGURE 8:**
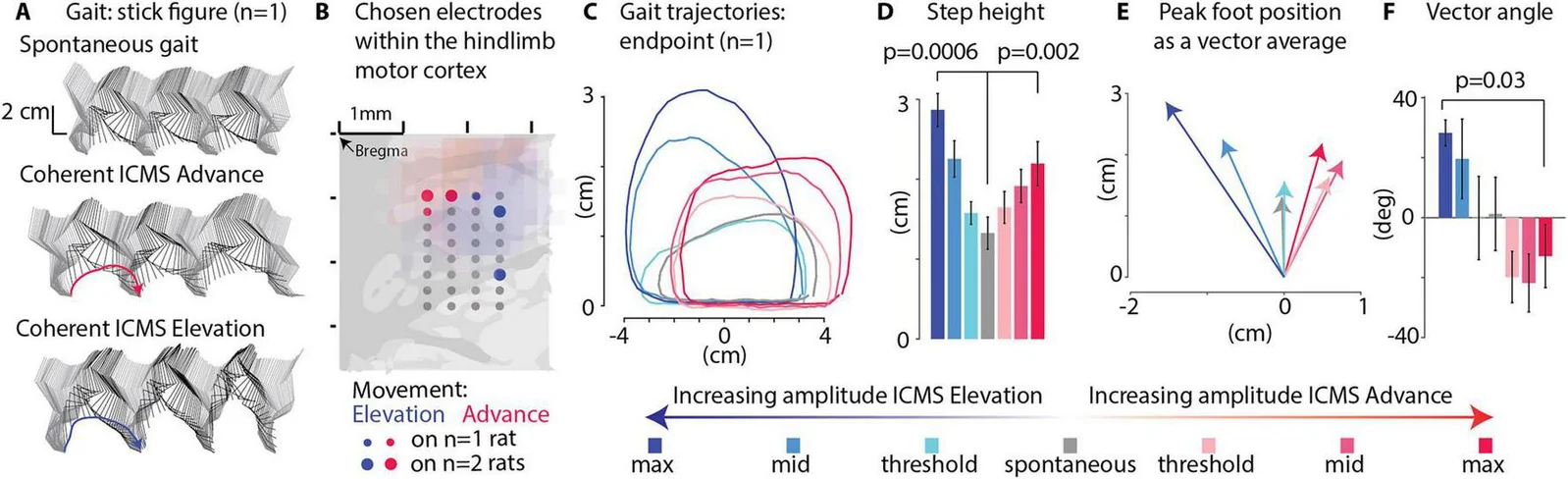
Intracortical stimulation of two hindlimb cortical subregions distinctly modulates gait kinematics. **(A)** Stick Figure representations of hindlimb kinematics during spontaneous locomotion (top), and during intracortical microstimulation (ICMS) applied at coherent phases. Stimulation was delivered using electrodes driving flexion-related movements either in the forward (middle) or backward (bottom) direction. **(B)** Electrode map of the hindlimb motor cortex showing stimulation sites eliciting backward (blue) and forward (red) flexion movements. Dots size indicates the number of animals (1 or 2) for which a given site was used. **(C)** Representative swing trajectories of the foot endpoint under increasing ICMS amplitude for forward (red hues) and backward (blue hues) flexion, as well as spontaneous gait (gray). **(D)** Quantification of step height across conditions, showing significant increases under maximal backward and forward ICMS (dark blue, dark red) compared to spontaneous gait and lower amplitudes (*N* = 5 rats). **(E)** Average vector representation of peak foot position during swing phase. **(F)** Mean vector angle relative to spontaneous gait (*n* = 5 rats). Bars represent mean ± s.e.m.

## Discussion

The motor cortex is essential for voluntary movement, and our findings using LD-ICMS in rats support the view that its organization reflects a repertoire of coordinated actions rather than a simple somatotopic map of individual muscles (Neafsey et al., 1986). Specifically, we identified two functionally distinct subregions in the hindlimb motor cortex that encode directionally specific movement types, Advance and Elevation, each characterized by unique kinematic signatures and spatial segregation. Phase-coherent stimulation in awake rats demonstrated the functional relevance of these subregions, selectively biasing hindlimb trajectories in a direction- and amplitude-dependent manner. These findings highlight the functional specialization of the hindlimb motor cortex and suggest promising cortical targets for neuroprosthetic strategies aimed at restoring locomotor control.

### Kinematics and typology: evidence for joint-synergy specialization

Our observation that LD-ICMS produces complex, multi-joint movements that converge to a final characteristic posture is consistent with prior studies on complex forelimb movements in rodents and primates (Bonazzi et al., 2013; Brown and Teskey, 2014; Graziano et al., 2002; Halley et al., 2020; Harrison et al., 2012). PCA objectively classified these movements, with PC1 capturing movement amplitude and PC2 capturing joint-pattern variability. Joint-level analyses confirmed this dissociation — Elevation exhibited faster mean and peak speeds at the knee and ankle, while Advance produced larger vector and trajectory lengths — indicating recruitment of functionally distinct joint synergies: a rapid upward-lifting program (Elevation) and a larger-amplitude, propulsion-oriented program (Advance). The independence of PC1 and PC2 suggests that the motor cortex modulates the vigor of a motor command separately from its type, consistent with prior demonstrations of independent control of muscle synergies (Bizzi and Cheung, 2013; Cheung et al., 2009; d’Avella and Bizzi, 2005; Holdefer and Miller, 2002; Morrow et al., 2009; Yakovenko et al., 2011), and parallels the organization of M1 population activity into separate low-dimensional subspaces for ongoing locomotor dynamics and volitional gait adjustments (Borgognon et al., 2025; Xing et al., 2022). The emergence of stereotyped multi-joint programs from localized cortical stimulation supports the concept that the motor cortex encodes coordinated motor primitives rather than isolated muscle twitches; the spatial substrate of this organization is examined in the following section.

### Stimulation parameters: duration versus intensity

Our parameter exploration revealed a clear dissociation in how train duration and intensity affect evoked movements. Specifically, increasing train duration converted simple muscle twitches into complex, multi-joint movements, consistent with prior studies showing that longer trains prolong motor-evoked potentials and recruit additional motor outputs (Brown and Teskey, 2014; Watson et al., 2016a,b).

In contrast, increasing intensity at a long duration primarily scaled the amplitude of the already engaged motor program without altering the movement type or joint pattern. Mechanistically, this suggests that LD-ICMS temporally recruits distributed cortical and spinal networks to assemble coordinated movement programs. In this framework, duration determines which motor program is engaged (recruitment), whereas intensity dictates the strength of its execution (amplitude). This dissociation provides a powerful axis for neurostimulation strategies, allowing for selective program activation with independently scalable output.

### Topography: link between kinematics and cortical localization

Advance and Elevation movements clustered in anteromedial and posterolateral subregions of the hindlimb motor cortex, respectively, with minimal overlap. Projection analyses along centroids revealed a strong correlation between PC2 scores and cortical location, providing quantitative evidence that kinematic differences align with spatial organization. While previous ICMS studies described clustering of complex forelimb movements in primates (Gharbawie et al., 2011; Graziano et al., 2002, 2005; Haiss and Schwarz, 2005; Overduin et al., 2012) and rodents (Bonazzi et al., 2013; Brown et al., 2023; Brown and Teskey, 2014; Halley et al., 2020; Harrison et al., 2012), most relied on qualitative visual observation. Our combined kinematic/PCA/topography design provides an objective demonstration that trajectory-defining kinematic features are spatially organized in the rat hindlimb motor cortex. Convergent evidence from intrinsic signal optical imaging combined with ICMS in non-human primates shows that task-related activity during reach-to-grasp is concentrated in patches overlapping less than 40% of the primary motor cortex and dorsal premotor cortex forelimb representations, with distinct spatiotemporal profiles between arm and hand zones, supporting functional subzones enriched for specific actions (Chehade and Gharbawie, 2023). Our findings extend this principle to the rodent hindlimb, providing the first quantitative topographic characterization of complex hindlimb movement representations.

### Functional recruitment and gait modulation

Phase-coherent stimulation during treadmill locomotion revealed that activating these subregions produces directionally specific changes in foot trajectories: a forward bias with Advance-region stimulation and greater step height/posterior shift with Elevation-region stimulation. These effects were direction-specific, amplitude-dependent, and reproducible across animals.

This extends our earlier demonstration that phase-coherent stimulation shapes hindlimb movements (Bonizzato and Martinez, 2021; Massai et al., 2023). While our prior study in cats showed a variety of evoked hindlimb movements using 100 ms ICMS trains (Duguay et al., 2023), mapping limitations prevented linking specific kinematic features to cortical topography. Here, we demonstrate that cortical subregions bias ongoing spinal locomotor patterns toward distinct end-effector trajectories, suggesting that the motor cortex issues high-level movement commands (direction and magnitude), while spinal circuits implement detailed inter-joint coordination, a scheme consistent with the organization of motor cortex population activity into distinct neural subspaces for baseline locomotion and volitional gait adjustments (Xing et al., 2022). The correspondence between movement programs identified under anesthesia and their directionally matched functional recovery in awake locomoting animals further suggests that LD-ICMS reveals genuine motor repertoire elements rather than anesthesia-induced artifacts, consistent with prior work showing that LD-ICMS evokes ethologically relevant postures conserved across consciousness states in primates (Graziano et al., 2002) and rodents (Brown and Teskey, 2014); the present study extends this principle to active hindlimb locomotion.

### Behavioral relevance

Although basic locomotion can be generated by subcortical and spinal networks (Barbeau and Rossignol, 1987; Musienko et al., 2012), the motor cortex is recruited for complex, precision-demanding tasks (Borgognon et al., 2025; DiGiovanna et al., 2016). The Advance and Elevation programs identified here resemble ethologically relevant maneuvers: a forward, propulsion-oriented adjustment consistent with the type of movement required for leading-foot obstacle clearing (Advance) and a rapid lifting maneuver consistent with a trailing-foot clearance (Elevation), consistent with the proposal that the motor cortex extends the robustness of subcortical movement systems specifically when stereotyped locomotor patterns must be modified by unexpected environmental demands (Lopes et al., 2023). This aligns with demonstrations in cats that the motor cortex modulates the phase and amplitude of sparse muscle synergies to reshape limb trajectories during obstacle stepping (Drew et al., 2008), and with recordings showing distinct motor cortex neural subspaces for basic locomotion and volitional gait adjustments (Xing et al., 2022). Phase-coherent stimulation in our awake preparation altered foot trajectories in directionally appropriate ways, suggesting these subregions may contribute to obstacle negotiation, precise foot placement on uneven terrain, or rapid step adjustments following perturbations. Future work should directly test whether Advance- and Elevation-region neurons are selectively recruited during obstacle avoidance, whether focal inactivation of each subregion produces the predicted directional deficit, and whether phase-coherent stimulation can assist gait in terrain-challenging or post-injury conditions.

### Limitations

Several limitations should be noted. First, ICMS mapping was performed under ketamine/xylazine anesthesia, which alters cortical excitability, movement thresholds, and extrapyramidal tract recruitment compared to awake conditions (Bonizzato and Martinez, 2021; Massai et al., 2023). Second, no EMG recordings were obtained during the anesthesia mapping sessions, precluding direct identification of the muscles differentially recruited by Advance versus Elevation programs. Future studies combining LD-ICMS with EMG will be needed to fully characterize the muscle synergy basis of these cortical programs.

Third, a fundamental interpretive challenge concerns the potential contribution of primary somatosensory cortex (S1) to the movements reported here. In rats, motor and somatosensory hindlimb representations may partially overlap, and the present study did not systematically localize electrode positions relative to their cytoarchitectonic border. This is directly relevant given that Halley et al. (2020) showed, using LD-ICMS across both areas in rats, that hip flexion moving the limb forward and hindlimb retraction were consistently evoked from S1, while hip extension driving the limb backward was observed from the motor cortex. Our Advance movement, characterized by hip and ankle flexion moving the limb forward, bears resemblance to movements evoked from S1 in that study, raising the possibility that some stimulation sites may have been in or near S1. Future work combining LD-ICMS with histological verification of electrode locations, or with reversible S1 deactivation during motor cortex stimulation, will be needed to fully disentangle the contributions of these two areas.

Fourth, long-duration, high-frequency stimulus trains have been criticized on the grounds that they may activate neural elements well beyond the stimulation locus through current spread (Strick, 2002) or through transsynaptic “neural hijacking” of interconnected cortical and subcortical networks (Cheney et al., 2013; Griffin et al., 2011). At the current intensities used here (150 μA), the estimated radius of direct excitation is approximately 0.34 mm (Stoney et al., 1968), which is small relative to the hindlimb map area (∼3.5 mm^2^); however, physiological spread via cortico-cortical and corticospinal axons could extend activation considerably further. Importantly, the spatially distinct topography of Advance and Elevation movements, the graded dose-response to stimulation intensity, and the functional similarity of movement programs in awake locomoting animals all argue against non-specific current spread as the primary driver of our observations. Nevertheless, transsynaptic recruitment likely contributes as an intermediate pathway, an inherent feature of any LD-ICMS paradigm.

Fifth, the exploration of stimulation parameters (Figure 2) was conducted at two representative sites, one per movement type. While the observed effects are consistent with prior studies (Brown and Teskey, 2014; Watson et al., 2016a,b), the generality of the duration-intensity dissociation across all cortical sites and movement types remains to be established with larger systematic sampling.

Sixth, a related interpretive concern is whether the evoked movements reflect nociceptive withdrawal rather than coordinated motor programs. Several observations argue against this interpretation: the graded, amplitude-dependent modulation of movement extent (Figure 2B) is inconsistent with an all-or-none nociceptive reflex; the directional specificity and topographic segregation of Advance and Elevation movements would not be expected from a non-specific pain withdrawal circuit; and the directionally matched recovery of these movement programs in awake, locomoting animals, where nociceptive confounds are minimized, further supports their identity as genuine motor repertoire elements. Nevertheless, definitive exclusion would require additional experiments such as targeted pharmacological blockade of nociceptive pathways, which we identify as a direction for future work.

### Clinical implications

The dissociation between program recruitment (duration) and amplitude (intensity), along with the reliable topography and high-level control properties of cortical stimulation, positions the hindlimb motor cortex as a promising target for cortical neuroprostheses aimed at gait restoration. Potential applications include:

1. Directional stimulation to bias foot placement during step initiation or swing phase.
2. Program selection with scalable amplitude (e.g., Elevation for clearance adjusted to obstacle height).
3. Closed-loop phase-coherent stimulation to deliver corrective commands during gait.

Such approaches may be particularly valuable following spinal cord injury, where spinal circuits can still execute synergy-level programs but require cortical input for adaptive control. However, SCI may restrict residual movement repertoires (Duguay et al., 2023), emphasizing the need to map remaining cortical outputs post-injury for effective neuroprosthetic implementation.

## Data Availability

The original contributions presented in this study are available in Supplementary Table 1 and are included in this article/Supplementary material, further inquiries can be directed to the corresponding author.

## Supplementary material

Supplementary Video 1 Immediate effect of phase-coherent cortical stimulation. During stimulation delivery, the LED at the bottom left of the screen is turned on. The rat first receives a set of forward-flexion ICMS, then a set of backward-flexion ICMS. Movie presented at 0.25× speed.
